# Aconite in Its Relation to the Throat

**Published:** 1890-03

**Authors:** 


### ACONITE IN ITS RELATION TO THE THROAT.

Throat in General.—Burning. Redness of soft palate and uvula. Scraping sensation in throat. Uvula swollen and enlarged; fauces injected, dark red. Pain, and difficulty in swallowing or in speaking. Fever, and bounding, full pulse.

Larynx and Trachea.—Laryngitis, with the inflammatory and mental symptoms peculiar to this remedy. Larynx is sensitive to touch and to inspired air, as if denuded. Complaints from overstraining the voice. (Also Alum., Arg., Arn., Arum-tr.)

Especially useful in croup, when caused by exposure to cold, dry winds. The patient awakes from first sleep; dry, short cough, but not much wheezing or sawing respiration. Loud breathing during expiration, every expiration ending in a hoarse, hacking cough. The patient is in an agony, is hot, restless, etc.

(Hepar has loose, rattling, croupy cough, worse after twelve P. M. Spongia has sawing, wheezing respiration between coughs. Acetic acid has loud breathing during inspiration.) The Aconite cough is dry and clear, or hoarse and hollow. It is worse after eating or drinking at night, lying on side; better lying on back (also Euphr., Mang.) Coughs after midnight, and the more he tries to suppress it the more severe it becomes. (Like Marum-verum.) A continuous short, hacking cough, with agonized tossing about; calls for Aconite (when the patient is *quiet*, study Squilla). Child grasps at the throat with each cough. (Also Ant-tart., and Allium-c. In croup Iodium and Lachesis; in delirium, Stramonium.) The respiration is labored, anxious, short, with the usual anxiety, restlessness, etc. In pleurisy the patient lies best on the back.

Generalities.—Aconite is mainly to be selected for any disease of the respiratory tract upon its general characteristics, which are very marked. It is never indicated in any patient who is quiet and serene, no matter how great the fever or the inflammation. For Aconite to relieve a fever or to stop any inflammatory process the patient must be restless, anxious, etc. The keynote of Aconite is fear, the patient is never cheerful or contented. Great fear, anxiety; and nervous excitability prevail as concomitants of all its symptoms. E. J. L.

